# Estimating the Respective Contributions of Human and Viral Genetic Variation to HIV Control

**DOI:** 10.1371/journal.pcbi.1005339

**Published:** 2017-02-09

**Authors:** István Bartha, Paul J. McLaren, Chanson Brumme, Richard Harrigan, Amalio Telenti, Jacques Fellay

**Affiliations:** 1 School of Life Sciences, École Polytechnique Fédérale de Lausanne, Lausanne, Switzerland; 2 Host-Pathogen Genomic Group, Swiss Institute of Bioinformatics, Lausanne, Switzerland; 3 National HIV and Retrovirology Laboratory, Public Health Agency of Canada, Winnipeg, Manitoba, Canada; 4 Department of Medical Microbiology and Infectious Diseases, University of Manitoba, Winnipeg, Manitoba, Canada; 5 BC Centre for Excellence in HIV/AIDS, University of British Columbia, Vancouver, British Columbia, Canada; 6 Genomic Medicine Group, J. Craig Venter Institute, La Jolla, California, United States of America; Eötvös Loránd University, HUNGARY

## Abstract

We evaluated the fraction of variation in HIV-1 set point viral load attributable to viral or human genetic factors by using joint host/pathogen genetic data from 541 HIV infected individuals. We show that viral genetic diversity explains 29% of the variation in viral load while host factors explain 8.4%. Using a joint model including both host and viral effects, we estimate a total of 30% heritability, indicating that most of the host effects are reflected in viral sequence variation.

## Author Summary

Viral loads of Human Immunodeficiency Virus infections are correlated between the donor and the recipient of the transmission pair. Similarly, human genetic factors may modulate viral load. In this study we estimate the extents to which viral load is heritable either via the viral genotype (from donor to recipient) or via the host’s Human Leukocyte Antigen (HLA) genotype. We find that a major fraction of inter individual variability is explained by the similarity of the viral genotypes, and that human genetic variation in the HLA region provide little additional explanatory power.

## Introduction

There are differences in the rate of disease progression among individuals infected with HIV. An easy to measure and reliable correlate of disease progression is the mean log viral load (HIV RNA copies per ml of plasma). The viral load measured during the chronic phase of infection (referred to as setpoint viral load, spVL) exhibits large variation in a population. Several studies have been carried out to elucidate whether this variation is primarily driven by host genetics [1–4], viral genetics [5–9], or environmental effects [7]. Genome-wide association studies consistently show that amino acid polymorphisms in the peptide binding groove of the HLA-A and HLA–B proteins are associated with the viral load of an individual. Furthermore, variants in the HLA-C and CCR5 genes have also been shown to impact spVL. However, those host factors explain less than 15% of the observed phenotypic variance [4]. In contrast, viral genetic studies and studies of donor-recipient transmission pairs established that 33% of the phenotypic variance is attributable to the transmitted virus itself [5, 10–13].

HIV is an extremely variable and adaptive organism with a rapid replication time, and high rates of mutation. Within-host evolution of the viral population occurs during the chronic phase of infection in which the pathogen adapts to its host environment. Several studies showed that a major proportion of the viral sequence is under selective pressure in the host environment, and several viral amino acid changes are associated with host genetic variants in the Human Leukocyte Antigen (HLA) genes [14, 15].

Viral strains harbor epitope sequences that can be presented by HLA class I proteins of the infected host, which allows the detection and killing of infected cells. The viral population evades detection through escape mutations that modify the epitope sequence but may incur a fitness cost. Compensatory mutations may follow until the viral population reaches its optimal place in a sequence space constrained by the host immune system [16].

There are two main different approaches to viral heritability estimation in the literature. The first one is based on the regression of phenotypic values in donor-recipient transmission pairs, while the other quantifies the difference between the observed phenotypic variance-covariance structure and the phylogenetic variance-covariance structure. Because our study population did not include donor-recipient data, we used the latter strategy. In particular we used linear mixed models (LMMs) to explain inter-patient differences in spVL while taking into account host and viral genetic relatedness. LMMs use the pairwise relatedness of individuals with respect to a large set of features (rather than the individual data points) to estimate the fraction of phenotypic variance attributable to those features. Such models have been successfully applied to estimate narrow-sense heritability from genome-wide genotype data [17]. Concurrently, LMMs were proposed to incorporate phylogenetic relatedness between samples in comparative analyses [18], a technique that was further developed to estimate the viral genetic contribution to spVL [6, 8].

## Results

To estimate the respective contribution of host and viral genetics to the variation in spontaneous HIV control, we collected paired viral/host genotypes along with spVL measurements from 541 chronically infected individuals enrolled in two prospective cohort studies in Switzerland and in Canada. We estimated the respective contributions of host and viral genetics to spVL by defining two relatedness measures, one with respect to the host genotypes, the other with respect to the viral genotypes, and used these jointly in a linear mixed model.

On the host side, we focused on amino acid variations in the HLA-A, B and C genes due to their established associations with HIV control [1]. In particular, we used 33 amino acid polymorphisms selected by L1 regularized regression [19] to represent the genetic relatedness of the host (**S1 Table**). Principal component analysis based on host genome-wide genotype data confirmed the lack of major population stratification in the host sample.

**Fig 1:**
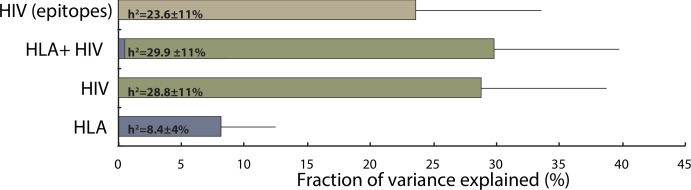
Illustration of fractions of explained variances by models taking human HLA, viral sequence similarities, or both into account.

We built three LMMs, one containing human variants, one derived from phylogenetic trees, and one including both host and virus information (**Fig 1**). The genetic relatedness matrix created from 33 amino acid polymorphisms of the human class I HLA genes explained 8.4% (SD = 4%) of the observed variance in spVL. In contrast, 28.8% (SD = 11%) of phenotypic variation was attributable to the viral phylogenetic tree. Combining the two relatedness matrices in one model yielded a total variance explained of 29.9% (SD = 12%), less than the sum of the latter two models. Thus, we show that HLA polymorphisms do not explain additional phenotypic variance beyond viral sequence variation.

We next assessed the contribution of viral variants most likely to have an impact on spVL. These included amino acids in known CTL epitopes [20] and those positions whose variation is associated with host polymorphisms [14] (82%, 60% and 84% of *gag*, *pol*, *nef* codons respectively, **S2 Table**). We used phylogenetic trees built from those codons to show that viral variation in epitopes or other HLA-associated positions explain 23.6% (SD = 11%) of phenotypic variance. However, this explained fraction might be overestimated due to linkage disequilibrium on the viral haplotype. We therefore repeated the analysis after randomly picking 70% of variable viral positions, and obtained very similar results. We thus cannot conclude that viral variants in known epitopes contribute disproportionately to variance in spVL. Additional evidence for the existence of substantial linkage disequilibrium on the viral haplotype comes from the analysis of the smaller, complementary set of variable viral positions (located in non-epitope regions), which explained 18.5% (SD = 10%) of the phenotypic variance. This leads to lower bounds of 11.4% and 6.3% of variance in spVL explained by variation in epitope and non-epitope regions, respectively, leaving 12.2% of variance unresolved due to linkage disequilibrium.

## Discussion

By jointly analyzing host and viral genetic relatedness, we here provide estimates of the total and respective contributions of human and viral genetic variation to HIV control. Our results do not challenge the current consensus estimates of the host or viral contributions to spVL. Nevertheless, our combined analysis demonstrates that human HLA polymorphisms do not explain additional variance in spVL once viral genetic diversity is taken into account.

The difference between the variance explained by viral phylogeny and the variance explained by HLA polymorphisms may be attributed to two effects. First, selected viral variants might provide a better surrogate of the impact of the host genotype than the imputed host amino acid variants we used. Rare host genetic factors outside of the major histocompatibility complex region (e.g. the *CCR5* deletion), as well as environmental interactions may influence viral fitness, and these effects are not accounted for in our estimate of host heritability. Thus some host effects might be missed from the host partition, while their footprint in the virus is still detected in the viral partition. Second, the difference could partly be due to the effect of viral variation independent of the current host, including transmitted escape mutations, i.e. viral sequence variation carried over from the previous host, rather than induced by the current host. Indeed, a recent study showed that spVL is dependent on the degree of pre-adaptation of the viral strain to the HLA class I genotype of the current host [21]. In particular, an increase in the frequency of pre-existing escape mutations, at the population level, led to higher viral heritability estimates. This indicates that both host and viral estimates of heritability depend on the amount of pre-adaptation in the sample population, which varies based on the level of HLA diversity. It has also been shown that reversion of some fitness reducing escape variants is very slow, potentially allowing for a transitory but measurable effect on viral load at the population level [15, 22].

A limitation of our study is the fact that study participants were collected from two cohorts. To reduce batch effect, we included a cohort-specific variable in all our models. Still, differences in inclusion criteria, health system, geographical exposure and other factors are very likely to increase environmental variance, thus negatively impacting our heritability estimates.

Another potential shortcoming is our implicit assumption of the absence of selection on spVL, which might be incorrect, as suggested by recent studies [23, 24], and might thus lead to over- or under-estimation of heritability due to model misspecification. Still, because our estimates are comparable to results obtained in donor-recipient transmission studies and in host-genetic studies, we conclude that they are useful for the purpose of delineating the respective amounts of host and viral contributions to phenotypic variation of HIV spVL.

In conclusion, our results suggest that host genetic association studies not taking the virus into account underestimate the population level effect of host genetic variation. Combining host and pathogen data provides additional insight into the genetic determinants of the clinical outcome of HIV infection, which can serve as a model for other chronic infectious diseases.

## Materials and Methods

### Ethics statement

All participants were HIV-1-infected adults, and written informed consent for genetic testing was obtained from all individuals as part of the original study in which they were enrolled. Ethical approval was obtained from institutional review boards for each of the respective contributing centers.

### Data collection

Bulk sequences of the HIV-1 *gag*, *pol* and *nef* genes, human genome-wide genotyping data and viral load measurements were obtained for 541 individuals of Western European ancestry infected with HIV-1 Subtype B, and followed in the Swiss HIV Cohort Study (SHCS, www.shcs.ch) or in the HAART Observational Medical Evaluation and Research study in Canada (HOMER, www.cfenet.ubc.ca/our-work/initiatives/homer) [14].

Viral sequences data were generated from samples collected two to five years after infection (for SHCS) or during chronic infection (for HOMER) but prior to the initiation of antiretroviral therapy. Thus, the viral genotypes reflect the result of natural adaptation of the pathogen to the host environment. The viral sequences for 1262, 2187 and 548 nucleotides of the *gag*, *pol* and *nef* genes were available for at least 80% of samples studied. The analysis was limited to these three genes because sequences of the rest of the retroviral genome were not available for the majority of study samples. Overlapping viral genomic regions were excluded from *gag*, to avoid duplicated sequences in the analysis.

Human DNA samples were genotyped in the context of previous genome-wide association studies. High-resolution HLA class I typing (4 digits; HLA-A, HLA-B, and HLA-C) was imputed from the genome-wide genotyping data as described previously [14].

Set point viral load (spVL) was defined as the average of the log10-transformed numbers of HIV-1 RNA copies per ml of plasma obtained in the absence of antiretroviral therapy, excluding VL measured in the first 6 months after seroconversion and during periods of advanced immunosuppression (i.e., with <100 CD4+ T cells per ul of blood). The distributions of spVL in the two cohorts are shown in **S1 Fig**.

### Viral genetic relatedness

The pairwise genetic relatedness of the dominant viral strains observed in the samples was calculated from phylogenetic trees similarly to [6]. Nucleotide sequences were translated to amino acid sequences, which were in turn aligned with MUSCLE [25] and used to derive the correct codon-aware nucleotide alignment. The phylogenetic tree was built from the aligned nucleotide sequences using RAxML [26] with the following command line: “raxml -w {PATH} -s {PATH} -m GTRCAT -f a -N 30 -k -n {NAME} -T {NUMBER} -x 1234 -p 1234”. Individual sequences were then rooted to the HIV-1 group M ancestral sequence, downloaded from the Los Alamos sequence database. Using an HIV-1 subtype C sequence as outgroup led to similar results. The whole tree was scaled with the inverse of the median height of the branches. We followed the method of Hodcroft et al, to create a relatedness matrix from a phylogenetic tree [6]. The genetic relatedness of two samples in a given phylogenetic tree is the amount of shared ancestry, i.e. the distance from the root of the tree (excluding the outgroup) to their most recent common ancestor [27].

### Host genetic relatedness

We selected 33 amino acid variants with L1-regularized regression (LASSO) out of all polymorphisms in the HLA-A, B and C genes and used them to generate a genetic relatedness matrix as described in [17]. Our relatively small sample size made it necessary to use a small subset of selected markers rather than genome-wide variant information to create the genetic relatedness matrix. Doing otherwise would have resulted in very large errors of the estimates.

### Heritability estimations

To estimate heritability, we used the gcta software as a generic implementation of the linear mixed model [17]. In such a framework, a multivariate Gaussian distribution models HIV viral load with a variance-covariance matrix consisting of the linear combination of the sample-sample genetic relatedness matrices (one for the host and one for the virus) and the identity matrix (representing sample-specific noise). The total heritability estimate is the fraction of variance explained by the genetic relatedness matrices over the total variance. All models included a binary variable indicating cohort as a fixed effect. Variance components were estimated by restricted maximum likelihood.

## Supporting Information

S1 Fig Distribution of HIV setpoint viral load values in the Swiss (SHCS) and Canadian (HOMER) cohorts. (PNG)

S1 Table List of human amino acid variants in HLA-I genes selected by L1 regularized regression and used throughout the paper. (XLSX)

S2 Table List of MHC-associated HIV amino acid positions based on epitope maps (20) and previous association studies (14). (TXT)
