# Supplementary figures and images for: Estimating the Respective Contributions of Human and Viral Genetic Variation to HIV Control
Source: PLoS Comput Biol. 2017 Feb 9;13(2):e1005339. doi: 10.1371/journal.pcbi.1005339 (PMC5300119; doi:10.1371/journal.pcbi.1005339)

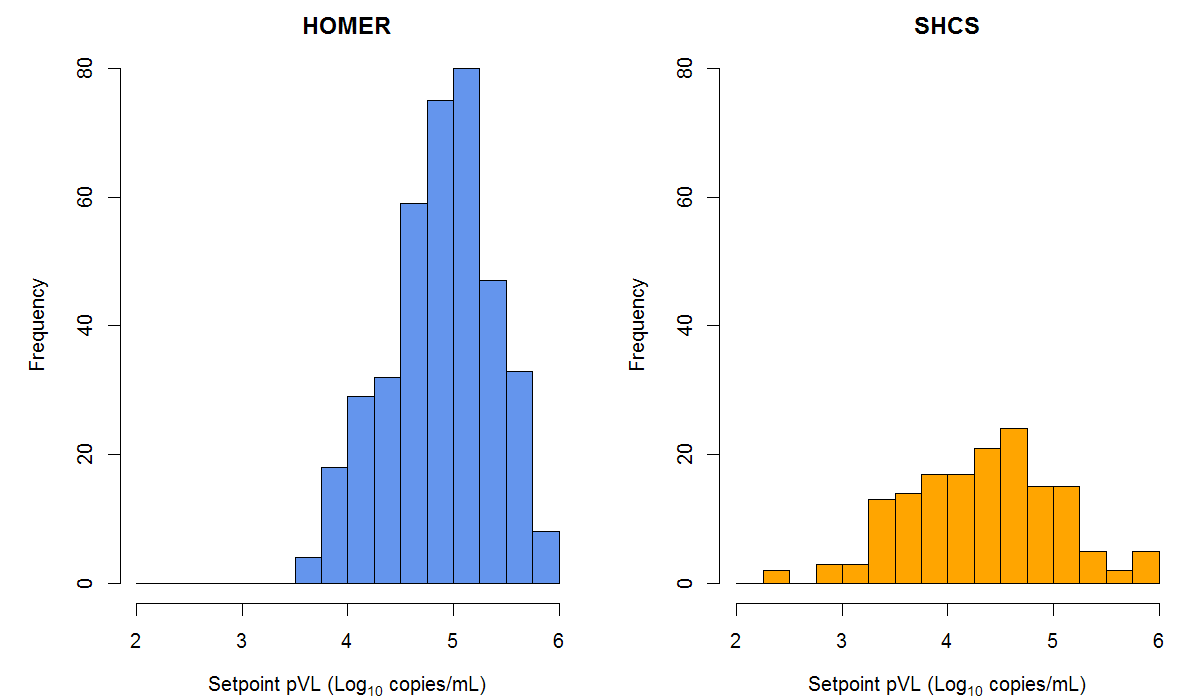

Supplement: S1 Fig — (PNG) [file pcbi.1005339.s001.png]
